# Healthy lifestyle linked to innate immunity and lipoprotein metabolism: a cross-sectional comparison using untargeted proteomics

**DOI:** 10.1038/s41598-023-44068-9

**Published:** 2023-10-04

**Authors:** David C. Nieman, Camila A. Sakaguchi, Matteo Pelleigrini, Michael J. Thompson, Susan Sumner, Qibin Zhang

**Affiliations:** 1 Human Performance Laboratory, Biology Department, Appalachian State University, North Carolina Research Campus, Kannapolis, NC USA; 2 UNCG Center for Translational Biomedical Research, University of North Carolina at Greensboro, North Carolina Research Campus, Kannapolis, NC USA; 3 Department of Molecular, Cell, and Developmental Biology, University of California Los Angeles, Los Angeles, CA USA; 4 Nutrition Research Institute, University of North Carolina at Chapel Hill, Kannapolis, NC 28081 USA

**Keywords:** Immunology, Physiology, Systems biology, Biomarkers, Risk factors

## Abstract

This study used untargeted proteomics to compare blood proteomic profiles in two groups of adults that differed widely in lifestyle habits. A total of 52 subjects in the lifestyle group (LIFE) (28 males, 24 females) and 52 in the control group (CON) (27 males, 25 females) participated in this cross-sectional study. Age, education level, marital status, and height did not differ significantly between LIFE and CON groups. The LIFE and CON groups differed markedly in body composition, physical activity patterns, dietary intake patterns, disease risk factor prevalence, blood measures of inflammation, triglycerides, HDL-cholesterol, glucose, and insulin, weight-adjusted leg/back and handgrip strength, and mood states. The proteomics analysis showed strong group differences for 39 of 725 proteins identified in dried blood spot samples. Of these, 18 were downregulated in the LIFE group and collectively indicated a lower innate immune activation signature. A total of 21 proteins were upregulated in the LIFE group and supported greater lipoprotein metabolism and HDL remodeling. Lifestyle-related habits and biomarkers were probed and the variance (> 50%) in proteomic profiles was best explained by group contrasts in indicators of adiposity. This cross-sectional study established that a relatively small number of proteins are associated with good lifestyle habits.

## Introduction

A healthy lifestyle has been associated in numerous epidemiological studies with increased life expectancy and decreased mortality rates for several types of chronic diseases^1–6^. Lifestyle models for these analyses typically included measures of body mass index (BMI), physical activity and diet intake patterns, smoking status, and alcohol intake, and results indicated that adherence to a collection of positive lifestyle habits was related to the lowest mortality rates.

Biologically relevant pathways related to health and lifestyle habits are being explored using proteomics, metabolomics, genetics and epigenetics, and other multiomic tools^7,8^. In a recent systematic review, Kaspy et al.^7^ urged that the identification of unique metabolomic profiles of combined healthy lifestyle behaviors may reveal novel biomarkers that would provide insights on the underlying mechanisms for primary prevention of chronic diseases. Proteomics involves the large-scale measurement of the structure and function of proteins and is useful in the identification of potential biomarkers for health, various disease processes, and treatment effects^9,10^. Previous studies focused on plasma proteome profiles associated with cardiorespiratory fitness status and acute and chronic exercise training^11–16^, the aging process^17,18^, disease prediction^19–21^, body composition, obesity, and weight loss^20–27^, and dietary intake patterns^24^.

Scientific understanding in this area of lifestyle habits and proteomics is emergent, and results from these studies are disparate and confusing due to different research designs and methods. A broad impact of adiposity on the human plasma proteome has been observed and most of these studies were population association studies^25^. None of these studies compared blood proteome profiles using a cross-sectional design with adults highly adherent or non-adherent to a multicomponent healthy lifestyle.

The purpose of this study was to use untargeted proteomics in comparing blood proteomic profiles in two groups of adults that differed widely in lifestyle habits. Study participants were recruited into lifestyle and control groups based on inclusion and exclusion criteria, and lifestyle habits and characteristics were measured using validated questionnaires and measurements of body composition and physical fitness in the Human Performance Lab. The goal was to identify a core list of proteins that were either upregulated or downregulated based on adherence to recommended lifestyle habits in adults. This lifestyle-related proteomic signature could be used in future clinical trials to determine the efficacy of various lifestyle and therapeutic interventions^9^.

## Results

A total of 52 subjects in the lifestyle group (LIFE) (28 males, 24 females) and 52 in the control group (CON) (27 males, 25 females) participated in this cross-sectional study (Table 1). The sex distribution did not differ between groups (*Χ*^2^ = 0.039, *p* = 0.844) and analyses were conducted for all study participants combined. A separate analysis (data not shown) showed that all group differences reported in this paper were significant when comparing males and females separately. The LIFE and CON groups were similar in basic demographic characteristics but differed markedly in body composition, physical activity patterns and physical fitness outcomes, dietary intake patterns, disease risk factor prevalence, and blood metabolic biomarkers.

**Table 1:** Subject and lifestyle characteristics, and related biomarkers.

Variable	Lifestyle (n = 52) (28 males, 24 females)	Control (n = 52) (27 males, 25 females)
Age (yrs)	47.5 ± 12.2	51.1 ± 10.5
Education (yrs)	16.3 ± 3.4	15.2 ± 3.1
Weight (kg)	67.8 ± 11.8*	101.5 ± 15.5
Height (cm)	172 ± 9.0	171 ± 9.5
Waist circumference (cm)	82.5 ± 7.5*	112.2 ± 11.5
Sagittal abdominal diameter (SAD) (cm)	17.5 ± 2.13*	26.9 ± 3.4
Body mass index (BMI) (kg/m^2^)	22.9 ± 2.6*	34.5 ± 4.0
Body fat (%)	22.6 ± 7.3*	40.8 ± 7.2
Fat mass index (fat mass kg/height m^2^)	5.32 ± 1.90*	14.2 ± 3.57
VO_2max_ (ml kg^−1^ min^−1^)	37.7 ± 8.8*	20.3 ± 8.4
Physical activity (MET-min/week)	5463 ± 3723*	2319 ± 2614
Leg/back strength (kg/kg body mass)	1.62 ± 0.6*	1.10 ± 0.5
Handgrip strength (sum right, left hands) (kg/kg body mass)	1.49 ± 0.6*	1.01 ± 0.5
Resting heart rate (RHR) (beats/min)	61.2 ± 10.1*	70.0 ± 10.5
Systolic blood pressure (sBP) (mm Hg)	115 ± 14.5	117 ± 14.5
Diastolic blood pressure (dBP) (mm Hg)	67.8 ± 10.0*	73.0 ± 10.5
Total mood disturbance (TMD)	90.4 ± 7.1*	94.8 ± 8.1
Fruit & vegetable (servings/day)	5.1 ± 2.0*	2.9 ± 1.4
Red meat (servings/day)	0.45 ± 0.64*	1.21 ± 0.72
Food nutrient index (FNI)	74.8 ± 15.8*	60.9 ± 15.8
Alcohol (grams/day)	10.2 ± 14.1	11.0 ± 20.0
Serum C-reactive protein (CRP) (mg/L)	0.77 ± 0.78*	2.61 ± 2.63
Serum insulin (U/L)	4.45 ± 2.7*	16.6 ± 12.6
Serum glucose (mg/dl)	86.8 ± 6.7*	99.8 ± 19.8
Homeostatic model assessment of insulin resistance (HOMA-IR)	0.97 ± 0.62*	4.23 ± 3.31
White blood cell count (10^9^/L)	4.8 ± 1.1*	5.8 ± 1.5
Serum cholesterol (mg/dl)	188 ± 31.5	188 ± 34.1
Serum low density lipoprotein (LDL) cholesterol (mg/dl)	106 ± 25.9	111 ± 30.1
Serum high density lipoprotein (HDL) cholesterol (mg/dl)	68.0 ± 17.5*	52.6 ± 17.9
Serum triglycerides (mg/dl)	75.3 ± 30.4*	141 ± 121
Serum bilirubin (mg/dl)	0.63 ± 0.31*	0.45 ± 0.20
Serum albumin (g/L)	4.61 ± 0.24*	4.41 ± 0.27
Serum carbon dioxide (mEq/L)	24.3 ± 1.7*	22.7 ± 2.6
Serum alkaline phosphatase (IU/L)	64.5 ± 16.7*	76.8 ± 27.3

Data expressed as mean ± standard deviation.

*p < 0.001, group difference.

Age, education level, and height did not differ significantly between LIFE and CON groups (Table 1). Race and ethnic backgrounds (LIFE, 88% white, 12% other; CON, 73% white, 27% other) did not differ significantly between groups (*Χ*^2^ = 6.86, *p* = 0.231). Group medical history comparisons for 41 past or current conditions showed significant differences for hypertension (LIFE 13%, CON 29%), arthritis (LIFE 4%, CON 21%), depression (LIFE 8%, CON 21%), sleep problems (LIFE 4%, CON 19%), and gallstones (LIFE 0%, CON 13%) (all *p* ≤ 0.05). Current medication use differed significantly between groups for beta-blockers/ace inhibitor hypertension drugs (LIFE 8%, CONs 23%), statin-based drugs (LIFE 8%, CONs 23%), depression drugs (LIFE 8%, CONs 21%), and metformin (LIFE 0%, CON 8%) (all *p* ≤ 0.05). None of the subjects in LIFE and only 2 in CON were current cigarette smokers, but 12% and 27% of LIFE and CON, respectively, reported smoking at least 100 cigarettes in their entire lifetimes (*Χ*^2^ = 3.96, *p* = 0.047). Body mass, waist circumference, body mass index (BMI), sagittal abdominal diameter (SAD), and body composition (body fat percentage and fat mass index (FMI)) differed significantly between the groups (*p* < 0.001) (Table 1).

Estimated VO_2max_ was 86% higher in LIFE versus CON (*p* < 0.001) (Table 1). Total physical activity at work, as part of house and yard work, to get from place to place, and during recreation, exercise, and sport was calculated as MET-min/week and was 136% higher in LIFE versus CON (*p* < 0.001) (Table 1). Weight-adjusted leg/back and handgrip dynamometer strength were significantly higher in LIFE versus CON (*p* < 0.001) (Table 1). Resting heart rate (RHR) and diastolic blood pressure (dBP) but not systolic blood pressure (sBP) was significantly lower in LIFE versus CON (*p* < 0.001) (Table 1). Total mood disturbance (TMD) calculated from the Profile of Mood States questionnaire (POMS) was modestly but significantly lower in LIFE versus CON (*p* = 0.004). Other questions related to stress and anxiety did reveal important group differences. Fruit and vegetable intake was higher and red meat intake lower in LIFE versus CON (both *p* < 0.001). The food nutrient index (FNI) calculated from 3-day food records and daily intake of eight micronutrients (vitamins A, C, D, E, folate, calcium, magnesium, potassium) was significantly higher in LIFE versus CON (*p* < 0.001) (Table 1).

Biomarkers including serum C-reactive protein (CRP) (− 70%), serum insulin (− 73%), serum glucose (− 13%), the homeostatic model assessment of insulin resistance (HOMA-IR) (− 77%), and serum triglycerides (− 47%), but not total serum cholesterol or LDL-cholesterol, were significantly lower in LIFE versus CON (all *p* < 0.001) (Table 1). HDL-cholesterol was 29% higher in LIFE versus CON (*p* < 0.001) (Table 1). Serum bilirubin, albumin, and carbon dioxide were significantly higher and serum alkaline phosphatase lower in LIFE versus CON (all *p* < 0.007). Other serum chemistries including blood urea nitrogen (BUN), creatinine, glomerular filtration rate (GFR), sodium, calcium, protein, and aspartate aminotransferase (AST) did not differ significantly between groups (data not shown). Serum thyroid stimulating hormone (TSH) and calcitriol (1,25-OH vitamin D) did not differ between groups (data not shown).

The untargeted proteomics analysis identified 970 proteins in at least one DBS sample, and 725 proteins in every sample. Principal component analysis (PCA) revealed outlier data from one study participant (LIFE group), and these data were removed from the proteomics analysis. Two-sample t-tests (LIFE vs. CON) with permutation-based FDR correction (Q < 0.05 alpha level), showed that blood levels of 39 proteins were found to be significantly different between groups. When the data were expressed as LIFE/CON group ratios, a total of 18 blood proteins were lower and 21 proteins were higher in LIFE versus CON. Protein descriptions and functions for the 39 proteins differing between the LIFE and CON groups are listed in Table 2.

**Table 2:** Descriptions and names of proteins that were lower (n = 18) and higher (n = 21) in the lifestyle versus control group (all 39 proteins, FDR correction, q < 0.05).

Group fold difference (log2)	Protein ID#	Gene	Protein names and short descriptions of functions
Proteins that were lower in the lifestyle versus control group			
− 0.5919	*P02743	APCS	*Serum amyloid P-component*; An acute phase protein involved in inflammation via complement-dependent pathways
− 0.441927	P00738	HP	*Haptoglobin*; Captures hemoglobin; acts as an antimicrobial and antioxidant and modulates the acute phase response
− 0.397979	*P00751	CFB	*Complement factor B*; Part of the alternate pathway of the complement system
− 0.362202	*P00740	F9	*Coagulation factor IX*; Participates in the intrinsic pathway of blood coagulation
− 0.327764	P0C0L5	C4B	*Complement C4-B*; Non-enzymatic component of C3 and C5 convertases; propagates the complement pathway
− 0.303047	P02763	ORM1	*Alpha-1-acid glycoprotein 1*; Transport protein; modulates immune activity during the acute-phase reaction
− 0.300494	*P01024	C3	*Complement C3*; Plays a central role in the activation of the complement system
− 0.286746	P05156	CFI	*Complement factor I*; Cleaves alpha-chains of C4b, C3b with cofactors C4-binding protein and factor H
− 0.286538	*P68104	EEF1A1	*Elongation factor 1-alpha 1*; Role in the positive regulation of interferon gamma transcription in T-helper 1 cells
− 0.282922	*P19652	ORM2	*Alpha-1-acid glycoprotein 2*; Transport protein; modulates immune activity during the acute-phase reaction
− 0.275192	P08603	CFH	*Complement factor H*; Functions as a cofactor in the inactivation of C3b
− 0.27405	P08311	CTSG	*Cathepsin G*; Cleaves complement C3 and has antibacterial activity
− 0.264261	*P43652	AFM	*Afamin*; Vitamin E binding protein
− 0.258812	P04004	VTN	*Vitronectin*; Cell adhesion factor for inhibition of the terminal cytolytic complement pathway
− 0.244408	P02751	FN1	*Fibronectin 1*; Involved in cell adhesion, cell motility, opsonization, wound healing, and maintenance of cell shape
− 0.229351	P05546	SERPIND1	*Heparin cofactor 2*; Thrombin inhibitor activated by the glycosaminoglycans, heparin or dermatan sulfate
− 0.202753	P36955	SERPINF1	*Pigment epithelium-derived factor*; Neurotrophic protein; induces neuronal differentiation; inhibits angiogenesis
− 0.132368	P01031	C5	*Complement C5*; Activation initiates the spontaneous assembly of the membrane attack complex
Proteins that were higher in the lifestyle versus control group			
0.105871	*Q15435	PPP1R7	*Protein phosphatase 1 regulatory subunit 7*; Regulatory subunit of protein phosphatase 1, and is required for completion of the mitotic cycle
0.129966	Q86X55	CARM1	*Histone-arginine methyltransferase CARM1*; Methylates guanidino nitrogens of arginyl residues and acts on histones and the regulation of gene expression
0.155758	P02647	APOA1	*Apolipoprotein A-I*; The major protein component of high density lipoprotein (HDL) in plasma. Participates in the reverse transport of cholesterol from tissues to the liver
0.160256	P06396	GSN	*Gelsolin*; Actin-modulating protein that is involved in filament assembly; influences cell processes including cells of the immune system
0.166127	P14550	AKR1A1	*Alcohol dehydrogenase [NADP( +)]*; Catalyzes the NADPH-dependent reduction of aldehydes to alcohols
0.175456	P01008	SERPINC1	*Antithrombin-III*; Most important serine protease inhibitor in plasma that regulates the blood coagulation cascade
0.188729	P08397	HMBS	*Porphobilinogen deaminase*; Produces an enzyme involved in heme production
0.194536	O94903	PLPBP	*Pyridoxal phosphate homeostasis protein*; Intracellular regulation of pyridoxal 5′-phosphate (PLP), vitamin B6
0.202922	*P02766	TTR	*Thyroid hormone-binding protein*; Transports thyroxine from the bloodstream to the brain
0.217191	*P37840	SNCA	*Alpha-synuclein*; Involved in the regulation of dopamine release and transport
0.228831	*P05089	ARG1	*Arginase-1*; Key element of the urea cycle converting L-arginine to urea and L-ornithine
0.244906	*P01023	A2M	*Alpha-2-macroglobulin*; Inhibits all four classes of proteinases by a unique 'trapping' mechanism. Inhibits inflammatory cytokines and inflammatory cascades
0.26744	*P02768	ALB	*Serum albumin*; Main function is the regulation of the colloidal osmotic pressure of blood; acts as a carrier protein for many molecules
0.267692	O95445	APOM	*Apolipoprotein M*; Involved with lipid transport
0.269348	P02654	APOC1	*Apolipoprotein C-I*; Plays a central role in HDL and very low density lipoprotein (VLDL) metabolism
0.273906	*P54252	ATXN3	*Ataxin-3*; Protein homeostasis maintenance, transcription, cytoskeleton regulation, myogenesis, and degradation
0.279698	P05090	APOD	*Apolipoprotein D*; Encodes a component of HDL; involved in lipoprotein metabolism and the transport and binding of bilin (e.g., bilirubin)
0.301995	*P35858	IGFALS	*Insulin-like growth factor-binding protein complex acid labile subunit*; Binds insulin-like growth factors, increasing their half-life; influences protein complexes, cell adhesion
0.405038	P01871	IGHM	*Immunoglobulin heavy constant mu*; Constant region of immunoglobulin heavy chains
0.411452	P04278	SHBG	*Sex hormone-binding globulin*; Androgen transport protein; regulates clearance rate of steroid hormones
0.415369	Q15848	ADIPOQ	*Adiponectin*; Adipokine that controls fat metabolism and insulin sensitivity, and anti-inflammatory activities

Group fold differences (lifestyle/control) are log2 transformed with minus values representing proteins downregulated in the lifestyle versus control group. “*” indicates the 15 proteins that were in common with the list of 20 proteins from multivariate modeling that predicted group membership. The 5 of 20 proteins included in the multivariate model that are not listed here were: P62877, Q9UNM6, Q13619, A4D1P6, Q9UBV8.

**Figure 1:**
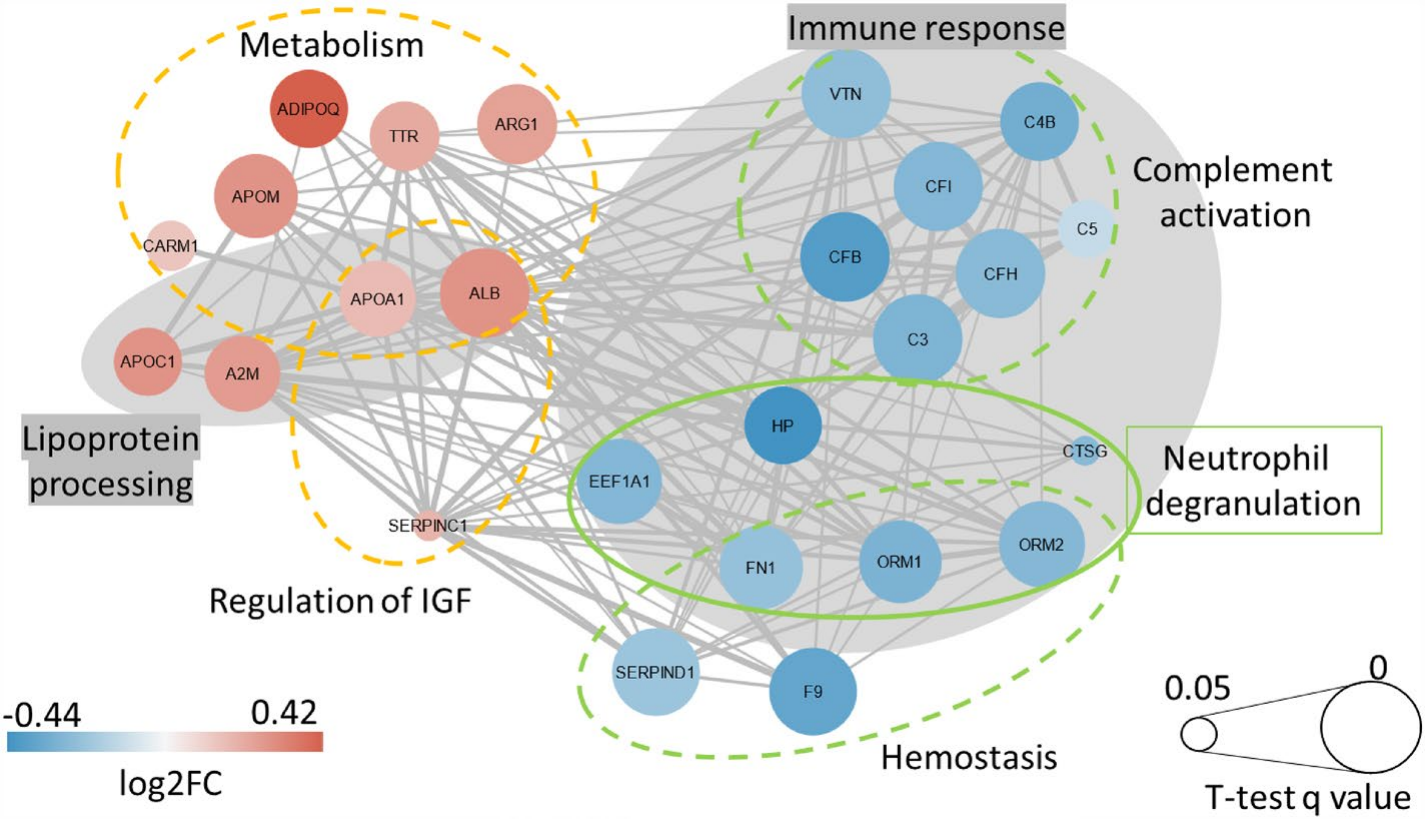
Protein–protein interaction network analysis with blue and red circles representing downregulated and upregulated proteins, respectively, for the lifestyle versus control group (log2 fold difference). The size of the circle represents the t-test q-value (larger circles have q-values closer to 0, and smaller circles closer to 0.05). The gray areas represent proteins downregulated in immune response activities and upregulated for lipoprotein processing. Other significant reactome pathways that differed between the lifestyle and control groups are indicated. This graph was developed with Cytoscape: Institute for Systems Biology. Cytoscape. 2023. Available from: https://www.cytoscape.org.

The 39 proteins were mapped onto STRING v11.5 to build protein–protein interaction (PPI) networks (http://string-db.org/) (Fig. 1). Figure 1 was developed with Cytoscape (Institute for Systems Biology. Cytoscape. 2023. Available from: https://www.cytoscape.org). A reactome pathway enrichment analysis showed that the primary pathways affected by group status were innate immune responses including complement activation and neutrophil degranulation that were lower in LIFE versus CON (all FDR < 0.001) (Fig. 1). The pathways most influenced by group status for the blood proteins that were higher in LIFE versus CON were plasma lipoprotein assembly, remodeling, and clearance, and HDL remodeling (all FDR < 0.025) (Fig. 1).

**Figure 2:**
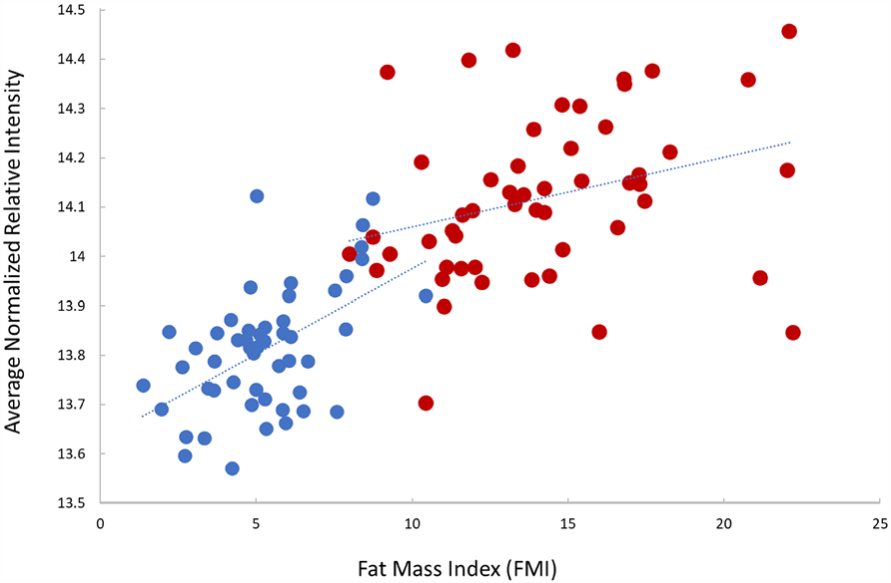
Scatterplot between the fat mass index (FMI) and the average normalized relative intensity for the 18 proteins that were lower in LIFE (blue circles) compared to CON (red circles). R^2^ = 0.587.

The normalized relative intensity data for the 18 blood proteins that were lower and the 21 proteins that were higher in the LIFE versus CON groups were averaged separately and then correlated with the variables in Table 1. The variables with the strongest correlations were included in backward elimination stepwise regression analyses. For the 18 blood proteins that were lower in LIFE versus CON, the model that emerged from stepwise regression included FMI and CRP [F = 75.5(2, 97), *p* < 0.001] (r^2^ = 0.609) (r^2^ = 0.587 for FMI alone). Figure 2 depicts the scatterplot for FMI and the average normalized relative intensity for the 18 proteins that were lower in LIFE versus CON. For the 21 blood proteins that were higher in LIFE versus CON, the best fit model included SAD, total white blood cell count (WBC), HDL-cholesterol, HOMA-IR, and the number of servings/day for fruits and vegetables [F = 56.4(5, 97), *p* < 0.001] (r^2^ = 0.744) (r^2^ = 0.52 for SAD alone).

**Figure 3:**
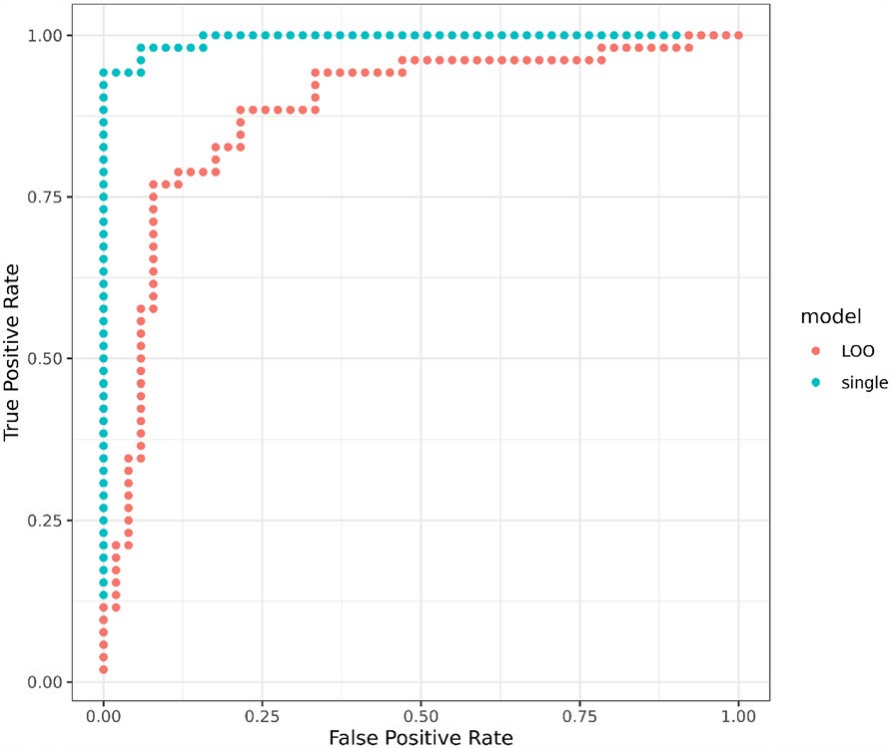
Receiver-operator-characteristic (ROC) curves for LIFE and CON group discriminators trained on the entire proteomics dataset. The blue curve is derived from category scores obtained from a single model optimized on all 103 samples, while the red curve depicts category scores obtained for each of 103 samples using 103 separate models optimized on 102 samples. LOO = “Leave-one-out” cross-validation.

A single group (LIFE and CON) discriminator was optimized over all 103 samples using logistic Lasso regression and a leave-one-out (LOO) iteration of the same was performed to get an estimate of how well a proteome-based discriminator might perform on new samples. From the logistic categorization scores obtained from these models, receiver-operator-characteristic (ROC) curves were plotted to illustrate the discrimination capabilities of these models. Results for both the single (over-fit) model and the LOO models are shown in Fig. 3. The area under the curves (AUC) for the single model and LOO models were 0.99 and 0.88, respectively, with *p* values of 2.6e−18 and 1.3e−11. The discriminator trained on all samples produced a multivariate model based on 20 proteins, and 15 of these were in common with the list of 39 proteins in Table 2 (noted with an “*”). The LOO iteration yielded LIFE versus CON group memberships that were 82% correct with a Fisher Exact Test *p* value of 6.5e−11.

## Discussion

The LIFE and CON groups differed markedly in body composition and fat mass-related anthropometric measurements, physical activity patterns and maximal aerobic fitness, dietary intake patterns, disease risk factor prevalence, blood measures of inflammation, triglycerides, HDL-cholesterol, glucose, and insulin, weight-adjusted leg/back and handgrip strength, and mood states. The proteomics analysis showed strong group differences for 39 of 725 proteins identified in the dried blood spot samples. Of these, 18 were downregulated in the LIFE group and collectively indicated a lower innate immune activation signature. A total of 21 proteins were upregulated in the LIFE group and supported greater lipoprotein metabolism and HDL remodeling. Lifestyle-related habits and biomarkers were probed and the variance (> 50%) in downregulated and upregulated proteins was best explained by group contrasts in indicators of body composition and visceral fat including FMI and SAD. Multivariate LOO modeling confirmed that group status (LIFE vs. CON) was strongly predicted by a proteomic signature consisting of just 20 proteins.

All but four of the 18 proteins downregulated in the LIFE versus CON group have immune-related functions. The elevation of proteins in CON indicates a systemic innate immune system activation consistent with higher serum CRP and metabolic inflammation^27–31^. Serum amyloid P (APCS), for example, is a pentraxin and an acute phase protein that along with CRP promotes phagocytosis via complement-dependent pathways^32^. Both SAP and CRP were substantially lower in the LIFE versus CON group along with nine other acute phase proteins including several complement proteins (C3, CFB, C4-B), alpha-1 acid glycoprotein 1 and 2 (ORM1, ORM2), haptoglobin (HP), vitronectin (VTN), and fibronectin 1 (FN1). These innate immune system-related proteins were most strongly associated with FMI, an indicator of high adiposity. Adipocytes express and excrete C3 and cohort studies show strong relationships of C3 with BMI and serum glucose and insulin^33,34^. Proteins that were strongly elevated in the LIFE versus CON group included adiponectin (ADIPOQ) and alpha-2 macroglobulin (A2M) that exert anti-inflammatory responses, and albumin (ALB) and thyroid hormone-binding protein (TTR) that are negative acute phase proteins. These proteins were strongly related to the abdominal sagittal diameter (SAD), a measure of visceral adiposity^35^. Other studies have linked obesity and weight change with CRP, APCS, ADIPOQ, C3 and other complement proteins, ORM1, and ORM2^22,34,36–38^. Many components in this network of proteins have been related to common chronic diseases including type 2 diabetes and cardiovascular disease^39^.

The reactome pathway analysis indicated that the LIFE versus CON group had lower levels of blood proteins related to innate immunity and neutrophil degranulation. Inflammation is an important component of innate immunity, but this process can become dysregulated with adiposity and is described as metabolic or systemic inflammation^27^. Metabolic inflammation is mediated through several types of immune cells including granulocytes, monocytes, and macrophages, and secreted proteins including acute phase proteins, proteases, cytokines/chemokines, and complement factors)^27–34^. Neutrophils can rapidly deploy protein enzymatic and chemical effectors, but this process must be tightly regulated to avoid inappropriate degranulation and tissue damage that can occur in many inflammatory and autoimmune conditions. Adipose tissue macrophages secrete many pro-inflammatory proteins that can promote insulin resistance^29^. Proteome and transcriptome studies of visceral adipose tissue in obese adults with type 2 diabetes have revealed upregulated proteins associated with innate immune system inflammation, dysregulated lipid metabolism, and complement activation, similar to the blood proteome profile of the CON group in the current study^31^.

The sex hormone-binding globulin (SHBG) was substantially higher in LIFE versus CON. Testosterone and estradiol circulate in humans bound to SHBG^40^. SHBG is negatively related to adiposity and serum glucose and insulin, but the relationship to inflammation has not yet been clearly established^36^. Low SHBG is a risk factor for type 2 diabetes and cardiovascular disease^41,42^. Thus, high SHBG combined with other proteins that were elevated in the LIFE group such as adiponectin (ADIPOQ), apolipoproteins A-1, M, C-1, and D, and alpha-2-macroglobulin (A2M) are consistent with a proteomic profile linked to lowered chronic disease risk^39,41,42^.

Several blood apolipoproteins were higher in LIFE versus CON including apolipoprotein A-1, D, and C-1 that are involved in HDL metabolism^43^. HDL-cholesterol was significantly higher in LIFE versus CON, and both lower body fat and higher physical activity levels have been related to elevated HDL-cholesterol^43,44^. Other studies have shown extensive effects of weight loss on apolipoproteins^22,36,38^.

Adiposity emerged as the single most important lifestyle-related factor in explaining the proteomic profile difference between LIFE and CON groups. Although physical activity and VO_2max_ did not survive modeling through stepwise linear regression, adiposity does represent a chronic imbalance between energy expenditure and intake, and thus is a strong indicator of nonadherence to recommended lifestyle habits. Plasma protein signatures linked to VO_2max_ and changes in cardiorespiratory fitness have been reported but only a few (insulin-like growth factor binding protein, gelsolin, and complement factor B) were common to our list of 39 proteins^12^.

There are several limitations in this cross-sectional study. Sample sizes were relatively small but statistically sufficient for a study of this type, and although group means for age did not differ, the age range was wide. Follow-up studies with larger sample sizes and narrower age ranges are recommended to confirm the findings of this study. Much of the data used in this study was acquired using self-reported questionnaires, and some responses (e.g., POMS, dietary intake, physical activity) were based on short time periods. These responses may not have accurately reflected the participants’ long-term lifestyle habits. Relatively small proportions of the subjects in both groups reported specific medical conditions and the use of medications, and the statistical models did not include these types of data but rather focused on the primary lifestyle habits. In general, this cross-sectional study provided a snapshot of LIFE versus CON group proteome differences at a specific point in time, and provided preliminary data that can be tested using more advanced study designs.

As demonstrated in this study, proteomics is an effective tool to explore the specific types of circulating proteins that are increased or decreased due to lifestyle habits in humans^45^. Our cross-sectional study of adults adherent and non-adherent to recommended lifestyle habits established strong group differences for 39 proteins primarily related to innate immunity and lipoprotein metabolism. Many of these protein differences were best explained by group contrasts in adiposity and visceral fat. The relatively small number of upregulated and downregulated proteins associated with good lifestyle habits should facilitate the development of a targeted “lifestyle” proteomic panel that can be used in future studies to determine the efficacy of various prevention and treatment strategies.

## Methods

### Study participants

Male and female study participants ages 25–75 years were recruited via mass advertising and targeted email messages to individuals in the Charlotte, NC, metropolitan area. Participants voluntarily signed the informed consent, and procedures were approved by the Appalachian State University Human Subjects Institutional Review Board (IRB), Federal Wide Assurance (FWA) number: FWA00027456. Notice of IRB approval by expedited review was granted by the IRB (#21-0054) on 10/16/2020. The research was performed in accordance with relevant guidelines and regulations, and informed consent was obtained from all study participants. All participants were healthy and noninstitutionalized, and able to provide written consent and follow verbal and written study directions in English. Participants were excluded if they were currently being treated for heart disease or cancer (excluding skin cancer), or medically complicated conditions (i.e., diabetes requiring insulin, uncontrolled high blood pressure). No restrictions were placed on diet, supplement usage, or common medications for hypertension, high blood cholesterol, diabetes (other than insulin), anxiety, depression, pain, gastrointestinal conditions, asthma, and hypothyroidism.

Other inclusion criteria for the lifestyle group were as follows: (1) Healthy, with no current history of chronic or infectious disease; (2) Not overweight or obese (body mass index less than 25 kg/m^2^); (3) High physical activity level (> 300 min per week, vigorous exercise); (4) Non-smoker for at least the previous three years; (5) Healthy dietary pattern (i.e., high intake of fruits, vegetables, whole grains, low-fat dairy products, healthy protein foods, and a low intake of salt, sugar, fats, and alcohol).

Other inclusion criteria for the control group were as follows: (1) Healthy, with no current history of chronic or infectious disease; (2) Obese (body mass index greater than or equal to 30 kg/m^2^); (3) Sedentary or low physical activity level (< 150 min per week); (4) Unhealthy dietary pattern.

Recruitment and scheduling for the study continued until n = 55 adults were identified that matched the inclusion and exclusion criteria for each group. Three participants in each group failed to complete study procedures and were dropped out of the study.

### Study design and methods

This study employed a cross-sectional design that compared metabolic and proteomic profiles in adults adhering (n = 52) or not adhering (n = 52) to lifestyle recommendations. Prior to the lab visit, participants received questionnaires via email with detailed instructions. Questionnaires included a medical health and lifestyle questionnaire, the International Physical Activity Questionnaire (IPAQ), and the Profile of Mood States (POMS). The short IPAQ questionnaire was used, and study participants answered questions about their physical activity patterns during the previous seven days^46^. The IPAQ scoring protocol was used to determine physical activity in terms of metabolic equivalent of task (MET)-minutes per week (MET level × minutes of activity/day × days per week). MET levels were set at 3.3 for walking, 4.0 for moderate intensity activity, and 8.0 for vigorous intensity activity. An abbreviated 40-item version of POMS was used, and participants rated moods using the “right now” approach^47^. All responses were based on a five-point scale anchored by “not at all” (score of 0) and “extremely” (score of 4). Scores for the seven subscales were calculated by summing the numerical ratings for items that contributed to each subscale, with the total mood disturbance (TMD) calculated by summing the totals for the negative subscales (tension, depression, anger, fatigue, confusion) and then subtracting the total for the positive subscales (vigor, esteem-related affect), and adding 100 to eliminate negative scores. The POMS TMD score was included in this study as a lifestyle index of mental health and wellbeing^35^. A VO_2max_ estimating equation was applied using age, body fat percentage (measured with BIA), and a physical activity ranking^48^.

Participants also received a 3-day food record with detailed instructions. Participants listed all foods and beverages consumed during a Thursday, Friday, Saturday time period prior to the lab visit. This food record was analyzed for micro- and macronutrient intake using the ESHA Food Processor (Version 11.11, ESHA Research, Salem, OR, United States). The nutrient data from the 3-day food records were used to calculate the food nutrition index (FNI)^49^. The FNI evaluates usual micronutrient intakes from foods and beverages relative to the recommended dietary allowance (RDA) or adequate intake (AI) standards for eight underconsumed micronutrients: calcium, magnesium, potassium, folate, and vitamins A, C, D, and E. The percentage of each micronutrient relative to the RDA or AI was truncated at 100% with each micronutrient weighted equally. The FNI overall score (ranging from 0 to 100) is the average of the component scores.

Participants reported to the lab at the scheduled appointment time in an overnight fasted state (i.e., no food, supplements, or beverages other than water for at least the previous 8 h). Participants sat down with the research staff and reviewed responses to questionnaires about lifestyle habits, physical activity levels, and mood states, and the 3-day food record.

After 10–15 min of seated rest, resting heart rate (RHR) and blood pressure were measured using the automated OMRON Digital Blood Pressure Monitor, HEM-907XL (OMRON Healthcare, Inc., Koyoto, Japan). Dried blood spot (DBS) specimens were collected via fingerprick onto standard blood spot cards (Whatman® protein saver cards, Sigma-Aldrich, St. Louis, MO, USA). One fingerprick provided 3–4 drops of blood and were dried at room temperature and stored at low humidity with desiccants.

A 35 ml blood sample was collected from an arm vein. Venous blood samples were collected in serum separation tubes (SST) and ethylenediaminetetraacetic acid (EDTA) containing blood collection tubes. SST were spun at 2300 rpm for 15 min after being allowed to clot for 15 min. Complete blood counts with white blood cell (WBC) differentials and serum samples were analyzed using Labcorp services (Burlington, NC) for these outcomes: comprehensive metabolic panel, lipid panel, thyroid stimulating hormone (TSH), calcitriol (1,25 di-OH vitamin D), C-reactive protein (CRP), and insulin. The Homeostatic model assessment of insulin resistance (HOMA-IR) was calculated from glucose and insulin data [(glucose mg/dl × insulin U/L)/405].

Participants were taken into the performance lab for measurements of height, weight, waist circumference, sagittal abdominal diameter, leg/back and hand grip dynamometer strength, and body fat (bioelectrical impedance or BIA)^35,50^. Height and weight (with BMI calculation) were measured using a seca stadiometer and weight scale (Hamburg, Germany). Waist circumference was measured at the level of the iliac crest with a seca measuring tape. The sagittal abdominal diameter (SAD) (anthropometric index of visceral adiposity) was measured using a Lafayette caliper (Lafayette Instruments, Lafayette, IN). SAD was measured at the height of the iliac crest with the participant in a supine position, knees bent, and feet flat on the examination table. Body composition was measured using the seca BIA Medical Body Composition Analyzer 514 bioelectrical impedance scale (Hamburg, Germany). Muscular strength was measured using a handgrip dynamometer and leg/back dynamometer (Lafayette Instruments, Lafayette, IN). The study participant applied chalk to each hand, with the dynamometer adjusted and placed comfortably in the hand to be tested. The participant assumed a slightly bent forward position, with the hand to be tested out in front of the body. The test involved an all-out gripping effort for 2–3 s. Each hand was tested 3 times, with the best score recorded for each hand and then summed. Leg/back strength was assessed with the legs slightly bent at the knee and participants grasping a bar attached via a chain to a force measuring device with straight arms, and then lifted up with maximal effort for several seconds. This was repeated three times with the best score recorded.

### Sample analysis

#### Proteomics

The DBS samples were used for the proteomics analysis^15,16^. DBS cards were center punched (4 mm) and added to two 96 wells plates in randomized order. Proteins were resolubilized from the punches in 8 M urea, 50 mM AmBic and 0.1 mM dithiothreitol (DTT) for 30 min at 37 °C while shaking. Proteins were subsequently alkylated using 0.1 mM iodoacetamide (IAA) for 30 min in the dark. Protein concentrations were measured. Samples were diluted 5 × with 50 mM AmBic to reduce the urea concentration to less than 2 M and proteins were digested at 37 °C using trypsin in a 1:50 trypsin to protein ratio. Data-independent acquisition mass spectrometry (DIA-MS) was performed on an Exploris 480 mass spectrometer (Thermo Scientific) coupled to a nanoLC (Dionex) with a flowrate of 300 nl/min for 60 min using a linear gradient. Data were acquired in a DIA mode in combination with BoxCar. For DIA MS2, 31 windows were created and optimized for DBS samples. MS1 resolution was set to 60,000 and MS2 resolution was set to 30,000. The MS1 fill time was 100 ms with automatic gain control (AGC) equal to 500%, and the MS2 fill time was 55 ms with AGC equal to 3000%. For BoxCar, MS1 windows were optimized for DBS samples for 3 × 10 boxes, with MS1 resolution set to 120,000 at a fill time set to 23 ms and AGC target equal to 100%. Identifications were performed against a customized library with 1012 proteins (pre-fractionated) and 11,791 peptides using an internal pipeline InfineQ 1.5 (ProteiQ Biosciences, Berlin, Germany) to increase protein coverage. Identifications were conducted while controlling for a 1% false discovery rate (FDR) with cross-run selection enabled and deep post-translational modification (PTM) analysis not enabled. Loess normalization was performed on peptide levels across all measurements. Spectra were analyzed for quality using Skyline^51^ with manual validation. Real time quality control of MS measurements was consistent showing stable LC–MS data acquisition and reproducible sample preparation. A total of 970 proteins were identified in at least one DBS sample, and 725 proteins were identified in every sample. Protein identification was based on LC–MS DIA BoxCar analysis with 5.8% missing values and a median technical coefficient of variation (CV) measured in technical replicates of 15.8%.

### Statistical analysis

The non-proteomics data are expressed as mean ± SD and were analyzed using SPSS (IBM SPSS Statistics, Version 28.0, IBM Corp, Armonk, NY, USA). Between group study participant characteristics were contrasted using independent t-tests for continuous data and Pearson’s chi-square for categorical data (Table 1). The Perseus computational platform was used for statistical analysis of the proteomics data^52^. Principal component analysis (PCA) was used to examine the potential for outlier samples in the proteomics dataset. Two-sample t-tests (LIFE vs. CON) with permutation-based FDR correction (Q < 0.05 alpha level) were used to probe for group contrasts in proteins. The proteins that were statistically different were mapped onto STRING v11.5 to build protein–protein interaction (PPI) networks (http://string-db.org/). A reactome pathway enrichment analysis was used to examine the primary pathways affected by group status. The normalized relative intensity data for the 18 blood proteins that were lower and the 21 proteins that were higher in the LIFE versus CON groups were averaged separately and then correlated with the variables in Table 1. The variables with the strongest correlations with these averaged values (cut-off *p* values ≤ 0.05) were included in backward elimination stepwise regression analyses using SPSS.

Group (LIFE, CON) discriminators based on proteomics data were constructed using penalized logistic regression. This analysis was conducted using the R packages “glmnet” (cran.r-project.org/web/packages/glmnet/)^53,54^ with the alpha parameter set to 1.0. This is equivalent to “Lasso” regression wherein the number of predictor variables in the categorization model is minimized. The normalized relative intensities for 725 proteins across 103 subjects were input into the algorithm with group status (LIFE, CON) as the binary category to be predicted. As it is well known that even penalized regression techniques will over-fit the training data when the number of predictor variables is much larger than the number of samples, a leave-one-out (LOO) protocol was used to get an estimate of how well a discriminator trained on this data might perform on new samples. The LOO approach consists of iterating over all N samples in the dataset. At each step one of the samples is withheld while a model is optimized over the other N-1 samples and a prediction is made for the sample that was held out.

## Data Availability

The datasets generated during and/or analyzed during the current study are available from the corresponding author on reasonable request. The mass spectrometry proteomics data have been deposited to the ProteomeXchange Consortium via the PRIDE partner repository with the dataset identifier PXD044448.
